# Iron Deficiency Prevalence in Bulgarian Children with Cerebral Palsy and Autism: A Call for Nutritional Interventions to Support Development

**DOI:** 10.3390/nu17121969

**Published:** 2025-06-10

**Authors:** Rositsa Chamova, Silviya Nikolova, Albena Toneva, Rozalina Braykova, Stanislava Hadzhieva, Yana Bocheva, Rouzha Pancheva

**Affiliations:** 1 Department of Hygiene and Epidemiology, Medical University, 9002 Varna, Bulgaria; albena_t@abv.bg (A.T.); rozalinabra@abv.bg (R.B.); slava790201@gmail.com (S.H.); rouzha.pancheva@gmail.com (R.P.); 2 NutriLect Research Group, Department of Neurosciences, Research Institute, Medical University, 9002 Varna, Bulgaria; silviya.p.nikolova@gmail.com; 3 Department of Social Medicine and Healthcare Organization, Medical University, 9002 Varna, Bulgaria; 4 Department of Clinical Laboratory, Faculty of Medicine, Medical University, 9002 Varna, Bulgaria; y_bocheva@yahoo.com

**Keywords:** autism, cerebral palsy, nutritional status, nutritional deficiencies, iron deficiency, serum ferritin, hemoglobin, anthropometry

## Abstract

**Background/Objectives**: Iron plays an important role in cognitive, behavioral, and motor development. This study aims to assess the iron nutritional status of Bulgarian children with cerebral palsy (CP) and autism spectrum disorder (ASD), focusing on iron deficiency (ID) and its impact on children’s development. We hypothesized that children with CP and ASD suffer from iron deficiency. **Methods**: The cross-sectional study includes 95 children from northeastern Bulgaria. Data were collected in two periods (2017–2018 and 2023–2024). Demographic questionnaires, food frequency questionnaires, and laboratory tests for hemoglobin, serum iron, serum ferritin, serum albumin, and CRP were conducted. Anthropometric measurements were evaluated. The Gross Motor Function Classification System scale was used to assess motor function in children with CP. Statistical analysis was performed using Jamovi software, ver. 2.6.44, with a significance level of *p* < 0.05. **Results**: Of the 95 children, 62.1% had CP and 37.9% had ASD. Most children had normal hemoglobin and serum iron levels, but 62.7% of those with CP and 36.8% of those with ASD had low serum ferritin levels, indicating latent ID. A higher proportion of children with CP than those with ASD consumed meat daily, while fish was more commonly consumed by children with ASD. Anthropometric data showed delayed growth and lower height-for-age scores in children with CP. **Conclusions**: The study identifies latent ID in children with CP and ASD. An evaluation of dietary habits highlights the need for interventions to improve nutritional status and development. The observed deficiencies emphasize the need for regular monitoring and targeted dietary programs for children in these groups.

## 1. Introduction

Iron is a crucial component of hemoglobin, as well as other proteins and enzymes that are vital for cell metabolism and overall survival. It also plays an important role in the development and functioning of various organs and systems. In the brain, for example, iron is involved in the myelination of white matter [1]. It is also important for neurotransmitters like dopamine, norepinephrine, and serotonin, which are essential for cognitive, behavioral, and motor development [2]. There is evidence supporting the critical role of iron in learning, attention, memory, and psychomotor functions [3]. Iron is stored in the liver as ferritin and hemosiderin and is transported between different body compartments by the protein transferrin [4]. Serum ferritin (SF) concentrations reflect iron stores and act as an early marker of iron deficiency (ID) [5]. SF is currently recommended as the most practical and universally available biomarker for detecting low iron levels [6]. According to the Food and Agriculture Organization of the United Nations (FAO), ID can be classified based on serum ferritin levels: depleted iron stores at SF < 24 ng/mL, mild ID at SF = 18–24 ng/mL, and severe ID at SF < 12 ng/mL [4].

Anemia in children is defined as hemoglobin (Hb) levels below 110 g/dL, and severe anemia as levels below 70 g/dL. The prevalence of anemia in children under 5 years old is estimated to be 18.1%. The prevalence of ID anemia varies by region, with 12.1% in Europe and 20.2% in Africa, where other factors also contribute [7]. Anemia is one of the most common nutritional diseases in children, with ID being the primary cause [8]. ID and ID anemia are more common in individuals with developmental and psychiatric disorders [9]. Inadequate dietary iron intake is a known cause of ID, and low iron intake has been linked to food selectivity, which is often observed in children with autism spectrum disorder (ASD) [10]. ID has been associated with an increased prevalence of neurodevelopmental disorders [11] and psychiatric conditions such as mood disorders, ASD, and attention deficit hyperactivity disorder [12].

Autism spectrum disorders are a group of highly heterogeneous conditions characterized by repetitive behaviors, difficulties in social interactions, and a wide range of neurodevelopmental and physical comorbidities [13]. In children with ASD, limited food preferences and picky eating habits are common, which can lead to decreased iron levels in this population [14]. Nutritional difficulties can negatively impact the child’s health [15], and severe malnutrition in children with ASD can result in stunted growth [5]. Restricted nutrition can also decrease iron absorption, leading to ID, which is more prevalent in preschool children with autism due to their limited food choices [16]. Several studies have reported decreased SF levels in autistic children from different populations [16,17,18].

Cerebral palsy (CP) is the most common pediatric motor disability globally, characterized by severe motor abnormalities, poor dietary intake, and impaired body composition [19]. Children and adolescents with CP are at a higher risk of malnutrition and micronutrient deficiencies [5], including ID. Nutritional deficiencies in these patients are not only evident through diet analysis but also through laboratory tests, which reveal lower iron levels compared to the healthy pediatric population [20]. ID is a significant issue that impairs cognitive, motor, and socioemotional abilities and is of particular concern in children with cerebral palsy [21].

We decided to check whether children with CP and ASD suffer from ID and how it correlates with anthropometric index Z-scores for weight-for-age (WAZ), height-for-age (HAZ), and body mass index-for-age (BMIAZ).

This study aims to investigate the iron status of Bulgarian children with ASD and CP, shedding light on the potential impact of ID on the specific developmental and health challenges faced by these groups.

This study is among the few in Bulgaria that have investigated the micronutrient status of children with neurodevelopmental disorders. The data on iron levels in children with ASD and CP can contribute to the broader epidemiological understanding established by studies conducted in Western countries.

## 2. Materials and Methods

### 2.1. Study Design

This cross-sectional study examines children with neurological conditions (CP, ASD) from northeastern Bulgaria—the cities of Varna and Ruse—from April 2017 to April 2018 (first period) and August 2023 to March 2024 (second period). The first phase served as a pilot descriptive study, while the second phase provided baseline data for a larger study investigating the relationship between dietary factors and bio-iron status. Both phases involved the measurement of bio-iron status and anthropometric data, including WAZ, HAZ, and BMIAZ, in children with ASD and CP. The current study involves the independent recruitment of participants across the two periods and does not include longitudinal follow-up. The data collected during both phases were compared to understand the trend and status of bio-iron and anthropometric markers across the two time periods. This comparison aims to provide insights into how these factors may evolve over time in children with ASD and CP.

### 2.2. Ethics

Recruitment began after obtaining approval from the Ethics Committee on Scientific Research of Medical University “Prof. Dr. P. Stoyanov”—Varna (protocols no. 60/23 February 2017 and no. 134/20 July 2023).

### 2.3. Participants and Recruitment

The researchers invited parents and guardians of children with CP and ASD attending centers of the Caritas Foundation (Varna), Equilibrium Association (Ruse), and the Varna Home for Medico-Social Care for Children through direct contact, phone calls, or emails. Semi-structured interviews were conducted to determine eligibility for participation, and informed consent was obtained from parents/guardians who agreed to participate in the study and follow up on their children’s health status. Confidentiality of personal information was ensured during data collection, processing, and storage.

#### 2.3.1. Inclusion Criteria

The study includes children from families residing in cities in northeastern Bulgaria, with an age range of 0–18 years during the first period and 2–12 years during the second period. Participants must have a confirmed diagnosis of cerebral palsy or autism spectrum disorder from a pediatric neurologist or pediatrician. Signed informed consent from parents/guardians is required for participation, along with the participation of parents in monitoring their children’s health status.

#### 2.3.2. Exclusion Criteria

Exclusion criteria include acute illness, severe life-threatening conditions, inborn genetic syndromes, difficulty understanding the study conditions, lack of proficiency in the Bulgarian language, and inaccessibility during the study period.

### 2.4. Study Measurements

The study aimed to collect and analyze data on the sociodemographic profile, dietary intake, and biochemical and anthropometric status of the children.

Sociodemographic data were collected using a questionnaire completed by parents, which included information on the child’s age, sex, ethnic origin, residence, parental education, employment status, and family structure.

Dietary intake was assessed through a food frequency questionnaire, which determined the frequency of meat and fish consumption as sources of easily absorbed iron.

The laboratory tests were performed in the clinical laboratory of the University Multi-profile Hospital for Active Treatment “St. Marina”—Varna. Blood sampling was conducted in the morning on an empty stomach to evaluate biochemical markers such as hemoglobin, serum iron (SI), and serum ferritin (SF), with reference ranges established at 112–146 g/L for Hb, 7.2–21.5 µmol/L for SI. The expected values for SF are men: 30,400 µg/L (ng/mL); women: 13,150 µg/L (ng/mL). Deficient states for these markers were defined as values below the lower limits of these ranges. CRP and serum albumin levels also were measured. The reference ranges for serum albumin are 32–48 g/L, and for CRP are 0–5.0 mg/L.

Anthropometric measurements, including weight and height, were obtained using standardized equipment and procedures. The children were dressed in light clothing and without shoes during the measurements. Weight and height were measured three times using a calibrated electronic scale and stadiometer (with an accuracy of 0.1 kg for body weight and 0.1 cm for height), and the average value was used for the analysis. When the child’s physical condition did not allow for direct measurement, the weight was calculated as follows: the parents were weighed in light clothing and without shoes, then weighed again while holding the child. The child’s weight was determined by subtracting the two values.

The Gross Motor Function Classification System scale was used to assess the overall motor activity of children with CP. The tool has five levels, with overall motor activity being least affected at level I and most affected at level V. In children with cerebral palsy and severe contractures (Gross Motor Function Classification System levels IV and V), height measurement was performed by measuring the length of the tibia with a Rigid Segmometer—Knee Height Caliper HOLWAY. For this purpose, the distance between the medial malleolus and the medial condyle of the tibia was measured three times, and the average value was used for the analysis. The patient’s knee and ankle were positioned at a 90-degree angle during the measurement. Height was calculated according to the formula proposed by Stevenson: Height (cm) = (3.26 × tibia length) + 30.8 ± 1.4 [22].

Anthropometric indices: WAZ, HAZ), and BMIAZ were calculated based on the World Health Organization growth standards [23].

### 2.5. Laboratory Methods

A colorimetric assay for the quantitative determination of iron in human serum on Cobas 6000 (Roche, Basel, Switzerland) was used. The method is based on the Ferrozine method without deproteinization. The electrochemiluminescence immunoassay “ECLIA” was used for the in vitro quantitative determination of ferritin in human serum. Cobas 6000 (Roche, Basel, Switzerland) was used. The cyanide-free SLS hemoglobin method on Sysmex XN 1000 (Sysmex Corporation, Kobe, Japan) was used for determination of hemoglobin levels in EDTA_K2 venous blood.

### 2.6. Statistical Analyses

To assess differences in the prevalence of nutritional deficiencies between the two groups of children, we used either a chi-square test or Fisher’s exact test. The chi-square test was employed for comparisons with adequate sample sizes, enabling an approximate evaluation of proportional differences. For comparisons involving smaller sample sizes, Fisher’s exact test provided an exact probability estimate, ensuring robustness in detecting statistically significant differences in deficiency rates. The statistical processing of data included methods to compare the results of children with CP and ASD across the two study periods. The methodology involved statistical tests to assess the significance of observed differences. For the analysis of nutritional deficiencies, Fisher’s exact test or the chi-square test was used to evaluate differences in the percentages of nutritional deficiencies between the two groups of children. Comparative analysis between the two periods was conducted using both the Mann–Whitney U test, a nonparametric test appropriate for comparing two independent samples with non-normal distributions, and an independent *t*-test, which were applied where assumptions of normality were met. To establish associations between anthropometric and hematological indicators, the Pearson correlation coefficient was used. Descriptive analyses were also performed, including frequency analysis to assess data distribution and arithmetic means to evaluate central tendency and dispersion. The analysis was based on complete-case analysis, where subjects with missing data were excluded from the analysis. All statistical analyses were conducted using SPSS v.23 and Jamovi software 2.6.44, with a significance threshold set at *p* < 0.05.

## 3. Results

### 3.1. Demographic Characteristics of the Participants

A total of 95 children with neuropsychiatric disorders were included in the study—63 in the first period and 32 in the second. Of these, 59 (62.1%) had a diagnosis of cerebral palsy and 36 (37.90%) had an autism spectrum disorder. In the first period, children with cerebral palsy made up 69.8% compared to 30.2% with ASD, while in the second period, those with ASD were more prevalent (53% compared to 47%). The mean age of the studied children was 6.37 ± 2.76 years. Of the total number of children participating in the study, 62.10% (*n* = 59) were male. There was a statistically significant difference in the distribution by gender among children with ASD, with 83.30% (*n* = 30) being male and 16.70% (*n* = 6) being female (χ^2^ = 11.1, *p* = 0.001). The mean age (years) of mothers of children with ASD and CP was 41.50 ± 7.88 and 45.55 ± 13.22, respectively (*p* = 0.22), and that of fathers was 42.16 ± 8.12 for children with ASD and 46.00 ± 12.17 for those with CP (*p* = 0.29). In terms of education, the highest relative proportion of mothers was those with a master’s degree (50.6%), while among fathers, those with a secondary education predominated (57.5%). The demographic characteristics of the participants in the study are presented in Table 1.

### 3.2. Hematological Parameters of the Study Sample

The distribution of children according to the values of hematological parameters in the first and second periods of the study is presented in Table 2. The analysis of hemoglobin and serum iron levels revealed that, in both study periods, the majority of children had normal values for these parameters. The difference between the proportion of children with normal and below-normal Hb levels was statistically significant (*p* = 0.001). In both study periods, one child (3.4%) was found to have low Hb levels, while 11.1% of the children had low SI levels in the first period, and 6.3% in the second period. Statistically significant differences were observed between the proportions of children with normal and below-normal SI levels in both study periods (*p* = 0.001).

When analyzing SF levels, it was found that the proportion of children with below-normal levels of this hematological marker was significantly higher in both study periods (62.70% vs. 37.30% for the first period; 62.50% vs. 37.50% for the second period) (*p* = 0.001). In both study periods, the relative proportion of children with normal serum albumin levels was significantly higher (Table 2).

The results indicate changes in the mean levels of hemoglobin, serum iron, and ferritin in the groups of patients with CP and ASD across the two different periods. In patients with CP, the mean Hb level was 126.0 ± 8.07 g/L in the first period and 132.8 ± 13.35 g/L in the second period. However, this difference was not statistically significant (t = −1.938, *p* = 0.06). Similarly, SI levels changed from 10.3 ± 4.22 µmol/L in the first period to 12.5 ± 4.04 µmol/L in the second period, but this difference was also not statistically significant (t = −1.52, *p* = 0.14). The mean SF levels showed minimal differences between the two periods (27.3 ± 19.56 ng/mL and 26.1 ± 17.78 ng/mL, respectively), with no statistical significance (t = 0.19, *p* = 0.85) (Table 3).

In patients with ASD, the difference between the mean hemoglobin value of 126.4 ± 5.41 g/L during the first study period and 128.6 ± 5.41 g/L during the second period was not statistically significant (t = −0.61, *p* = 0.55). The mean SI levels were 12.8 ± 4.82 µmol/L in the first period and 13.3 ± 3.63 µmol/L in the second period, with no significant difference (t = −0.26, *p* = 0.79). The mean SF levels also did not show a significant difference, with values of 34.40 ± 11.89 ng/mL and 37.60 ± 24.16 ng/mL for the first and second periods, respectively (t = −0.26, *p* = 0.80) (Table 4).

### 3.3. Food Consumption

Group differences in meat and fish consumption frequencies between children with cerebral palsy (CP) and those with autism spectrum disorder (ASD) were examined separately for the two study periods. In the first period, meat consumption was higher among children with ASD (mean = 5.86, SD = 1.86) compared to those with CP (mean = 4.70, SD = 2.06) (U = 86.5, *p* = 0.070). For fish consumption during the same period, children with CP reported a higher intake (mean = 1.33, SD = 0.78) than those with ASD (mean = 0.71, SD = 0.76), again with a marginally non-significant difference (U = 88.5, *p* = 0.065). In the second period, there was no significant difference in meat consumption between the CP group (mean = 4.40, SD = 1.51) and the ASD group (mean = 3.86, SD = 1.83; U = 55.5, *p* = 0.369). However, a statistically significant difference in fish consumption was observed, with children with CP consuming fish more frequently (mean = 5.00, SD = 0.00) compared to those with ASD (mean = 2.71, SD = 1.82; U = 27.5, *p* = 0.002) (Table 5).

### 3.4. Anthropometric Measurements

The anthropometric measurements for children with CP and ASD are presented in Table 5 and Table 6, respectively. Analyzing the results for children with cerebral palsy across the two study periods, we observed a statistically significant improvement only in HAZ, with the mean value increasing from −3.12 in the first period to −1.80 in the second period (*p* = 0.04), indicating substantial growth progress. WAZ showed an improvement from −2.96 to −2.11, but this change was not statistically significant (*p* = 0.17). Similarly, BMIAZ exhibited a slight change from −2.04 to −1.34, which also lacked statistical significance (*p* = 0.31) (Table 6).

When analyzing the physical development data for children with ASD, comparisons between the two study periods reveal that the mean WAZ decreased from 0.3850 (SD = 1.02) in the first period to 0.00533 (SD = 1.28) in the second period, but this change was not statistically significant (*p* = 0.37). HAZ also showed a decrease from 0.5941 (SD = 0.89) to −0.05133 (SD = 1.39), with no statistically significant difference (*p* = 0.13). Additionally, BMIAZ remained nearly unchanged, moving from 0.0206 (SD = 1.27) in the first period to −0.04867 (SD = 1.31) in the second period, and this difference was not statistically significant (*p* = 0.88) (Table 7).

### 3.5. Correlation Analysis of Nutritional and Biochemical Indicators

Correlation analysis reveals significant relationships between various nutritional and biochemical indicators. WAZ is strongly positively correlated with HAZ (r = 0.85, *p* < 0.001) and BMIAZ (r = 0.77, *p* < 0.001). Serum iron shows a positive correlation with WAZ (r = 0.31, *p* = 0.04) and HAZ (r = 0.33, *p* = 0.03), while ferritin is positively related to serum iron (r = 0.26, *p* = 0.06). Hemoglobin is significantly correlated with BMIAZ (r = 0.45, *p* = 0.002) and serum iron (r = 0.32, *p* = 0.03). Albumin exhibits strong positive correlations with WAZ (r = 0.43, *p* = 0.003), HAZ (r = 0.37, *p* = 0.008), and BMIAZ (r = 0.37, *p* = 0.010) (Table 8).

## 4. Discussion

The present study examined the iron nutritional status of children with CP and ASD. The specific eating patterns of these children, such as food aversions or preferences for certain types of food, put them at risk of nutritional deficiencies. This study identified the presence of latent ID in the examined group, which could potentially affect both their health and neuropsychological development. The results confirmed the hypothesis, namely, that children with CP and ASD suffer from ID.

Importantly, while several comparisons between the two study periods reached statistical significance, it is important to note that some of the absolute differences—particularly in hemoglobin, serum iron, and serum albumin levels—were relatively modest and remained within normal reference ranges. These changes, although statistically significant, may have limited clinical relevance. Given the descriptive nature of the study and the sample size, the findings should be interpreted as indicative of potential trends rather than definitive clinical shifts. Future research with larger cohorts and longitudinal follow-up is needed to clarify whether these differences have meaningful health implications.

Our analysis of hematological indicators, including hemoglobin, serum iron, and ferritin, reveals that despite the predominance of normal values for hemoglobin and serum iron, a higher proportion of children have below-normal serum ferritin levels. Serum ferritin is a key marker for assessing iron stores, and low levels can indicate ID or anemia [10]. Serum ferritin concentrations reflect iron deposits, acting as an early marker of ID [24].

SF has certain limitations as an indicator of ID. Since SF also acts as an acute-phase protein, systemic inflammation can lead to elevated levels of it. During the inflammatory response, serum levels of acute-phase proteins, including ferritin, C-reactive protein, and alpha-1-acid glycoprotein, rise significantly due to the increased expression of cytokines such as IL-6 [25]. The simultaneous measurement of C-reactive protein and serum ferritin increases the reliability of SF as an indicator of ID. We measured CRP levels to assess the role of inflammation, and all participants were found to have normal CRP levels, indicating that inflammation was not a contributing factor in this cohort.

Statistical comparisons showed no significant differences (*p* > 0.05) between periods, indicating persistent nutritional deficiencies without significant changes. This underscores the need for ongoing monitoring and appropriate interventions to improve nutritional status and overall well-being in this vulnerable group. Other studies also observe trends of ID in children with ASD and CP [9,24,26,27,28].

An important finding of this study concerns the dietary intake patterns of children with cerebral palsy and autism spectrum disorder, particularly in relation to the consumption of meat and fish—both key sources of bioavailable iron and essential nutrients. While meat consumption remained relatively consistent across the two study periods, a clear distinction emerged between the groups. Children with ASD exhibited a tendency toward frequent meat intake, with a high proportion consuming it five times a week or more. This pattern is consistent with previous observations suggesting increased meat consumption in children with ASD, possibly linked to sensory preferences or behavioral rigidity [29]. National dietary data indicate that while approximately half of Bulgarian children aged 1–9 years consume meat within the recommended range of 2–4 times per week, intake among school-aged children tends to be lower [30]. In our study, children with CP showed a more moderate meat intake pattern, which may reflect different feeding challenges such as oral-motor difficulties or spasticity that can affect dietary variety and volume.

In contrast, fish consumption revealed a dynamic shift over time, particularly among children with CP. In the initial period, fish intake was low in both groups, and a large proportion of CP children did not consume fish at all. However, by the second period, there was a substantial increase in fish consumption among children with CP, aligning more closely with nutritional recommendations. This change coincided with improvements in weight-for-age z-scores, suggesting that enhanced dietary quality—particularly increased intake of iron- and omega-3-rich fish—may have contributed to better growth outcomes. Previous studies have highlighted inadequate fish intake among children with ASD, often attributed to sensory aversions and food selectivity [31,32], and our findings are consistent with this literature. National survey data show that 80% of Bulgarian children meet the guideline of consuming fish at least once per week [30], yet in our study, few children with ASD achieved this target, and only a small minority consumed fish twice per week as recommended.

The most common cause of ID is the inadequate dietary intake of sources of easily absorbable iron. In children with autism, this is attributed to food selectivity and preferences for specific smells, colors, texture, and food tastes. Another reason for ID could be the frequent gastrointestinal problems in these children, which may affect iron absorbtion [11,14]. Feeding difficulties due to dysphagia and spasticity, and the type of food, contribute to energy and micronutrient deficiencies in children with CP [24]. Additionally, the increased effort or stress involved in oral feeding can be a significant challenge for these children [33]. Our focus on dietary protein sources, such as meat and fish, stems from their role as key providers of bioavailable heme iron. Heme iron is less affected by dietary inhibitors and is absorbed more efficiently than non-heme iron, making these foods critical in addressing iron deficiency, particularly in children with limited dietary diversity. Additionally, protein-rich foods provide other essential nutrients, such as vitamin B12 and zinc, which contribute to overall growth and development.

In our study, WAZ and HAZ showed a strong positive correlation with BMIAZ, and serum iron was positively correlated with WAZ and HAZ. A possible explanation is that the positive correlations between serum iron levels and anthropometric indices (WAZ, HAZ) may reflect the influence of adequate iron stores on growth. Iron is essential for metabolic processes, including cellular energy production and oxygen transport, which are critical for growth. Gerasimidis (2022) notes that serum albumin and protein levels are unreliable markers of nutritional status due to their sensitivity to hydration and inflammatory states rather than dietary intake [34]. However, we found that albumin is positively correlated with WAZ, HAZ, and BMIAZ.

The weak or non-significant correlation between serum ferritin and anthropometric indices may be attributed to ferritin’s role in reflecting oxidative stress and its complex relationship with the severity of malnutrition. The effect of puberty on growth and iron metabolism may contribute to variability in these correlations. Adolescents experiencing pubertal growth spurts often require higher iron intake, which could influence the observed relationships. Additionally, hormonal changes during puberty may impact hematological parameters.

The normal CRP levels further confirm that inflammation was not a confounding factor, underscoring the need for alternative, more direct indicators such as serum ferritin and anthropometric measures.

Nutritional status is essential for assessing the health and development of children with disabilities, as it influences both physical and neuropsychological outcomes. Poor nutrition can impair growth, motor function, bone health, and social adaptation [35]. Studies show that children with cerebral palsy are more often underweight, while those with autism are more likely to be overweight or obese [36].

Our study shows that children with CP are more likely to have very low (in the first period) or below-normal weight (in the second period) based on the WAZ indicator, while children with ASD predominantly have normal weight (WAZ medians in the first and second periods were 0.06 and 0.08, respectively). The change in WAZ, HAZ, and BMIAZ values in the second period for children with CP suggests that interventions or conditions during the observation period positively affected linear growth, though effects on weight and BMI were insufficient for significant changes. Monitoring anthropometric indicators in children with ASD revealed no significant changes in weight, height, or BMI-for-age across the periods examined.

Iron deficiency disrupts dopamine metabolism and affects brain regions essential for memory, attention, and learning, such as the hippocampus and corpus striatum [37,38,39]. It is linked to neurocognitive impairments and increased behavioral disorders [39]. For example, individuals with ID perform worse on tasks requiring cognitive control due to disrupted dopamine signaling [37]. Early ID may have lasting effects, including altered brain connectivity and reduced attention [38], highlighting the need for timely intervention. Beyond iron, factors like sleep hygiene and overall nutrition also influence cognitive outcomes, and should be addressed alongside supplementation [14,33,39].

The findings of this study, which highlight a high prevalence of latent ID among children with CP and ASD, align with this body of evidence. The potential for ID to exacerbate developmental challenges in these populations underscores the importance of targeted nutritional interventions, including dietary strategies to improve iron intake and address broader nutritional needs.

### Limitations

This study has several limitations. Firstly, the absence of a control group restricts the ability to compare findings with a healthy population, potentially affecting the interpretation of the results. Secondly, the cross-sectional nature of the study, coupled with the relatively small sample size, may limit the generalizability of these correlations. The cross-sectional methodology does not allow for the assessment of the duration of ID in the studied population. The research was conducted in a geographically limited area, which may not fully represent the broader population, potentially affecting the applicability of the results to other regions or settings. Additionally, the absence of a control group and reliance on single-point measurements could contribute to variability in the observed relationships. Furthermore, potential biases in participant selection and the reliance on self-reported dietary data could introduce inaccuracies and affect the validity of the findings. The intake of vitamins and medications for the treatment of diagnosed iron deficiency was not discussed in the present study.

This study primarily focused on dietary factors, such as protein and iron intake, as contributors to anemia and ID. Other potential causes, such as chronic blood loss, parasitic infections, and gastrointestinal malabsorption, were not explored. Investigating these factors in future studies would provide a more comprehensive understanding of anemia’s multifactorial etiology in children with CP and ASD. Despite its limitations, this study provides important preliminary data and identifies key patterns that can serve as a foundation for future longitudinal studies and research aimed at uncovering the biological mechanisms underlying iron deficiency in children with CP and ASD.

## 5. Conclusions

Latent iron deficiency has been identified among the studied groups of children. By evaluating their dietary habits and nutritional status, nutritional interventions aimed at preventing deficiencies and improving their physical and neuropsychological development as well as overall health would be possible. Further discussion is needed to connect the findings on iron deficiency with its potential negative impact on developmental outcomes.

## Figures and Tables

**Table 1:** Demographic and socioeconomic characteristics of children with CP and ASD.

	N (%)
Gender of children	
Male	59 (62.1%)
Female	36 (37.9%)
Diagnosis of children	
CP	59 (62.1%)
Autism	36 (37.9%)
Marital Status of Parents	
Married	54 (68.4%)
Cohabiting without marriage	15 (19%)
Divorced	4 (5.1%)
Never married	4 (5.1%)
Widowed	1 (1.3%)
Separated without divorce	1 (1.3%)
Employment Status of the Mother	
Full-time employment	64 (73.6%)
Temporary employment	2 (2.3%)
Unemployed	7 (8%)
Housewife	12 (13.8%)
Student	1 (1.1%)
Not working due to disability	1 (1.1%)
Employment Status of the Father	
Full-time employment	67 (89.3%)
Temporary employment	2 (2.7%)
Unemployed	4 (5.3%)
Retired	2 (2.7%)
Education Level of the Mother	
Primary education	3 (3.4%)
Secondary education	32 (36.8%)
Vocational secondary education	2 (2.3%)
Bachelor’s degree	6 (6.8%)
Master’s degree	44 (50.6%)
Education Level of the Father	
Primary education	1 (1.4%)
Secondary education	42 (57.5%)
Vocational secondary education	4 (5.5%)
Bachelor’s degree	25 (34.2%)
Doctoral degree	1 (1.4%)

**Table 2:** Distribution of children according to the values of the hematological parameters in the first and second periods of the study.

	First Period		Second Period	
Indicator	N, % [95%CI]	*p*-Level	N, % [95%CI]	*p*-Level
Hemoglobin				
Below norm	1/29, 3.4% [0.0008–0.178]	0.020	1/29, 3.4% [0.0008–0.178]	0.020
Norm	27/29, 93.1% [0.771–0.992]	0.001	27/29, 93.1% [0.772–0.992]	0.001
Above norm	-		1/29, 3.4% [0.0008–0.178]	0.020
Serum Iron				
Below norm	6/54, 11.1% [0.041–0.226]	0.002	2/32, 6.3% [0.0076–0.208]	0.002
Norm	48/54, 88.9% [0.773–0.958]	0.001	30/32, 93.8% [0.791–0.992]	0.001
Ferritin				
Below norm	37/59, 62.7% [0.491–0.750]	0.001	20/32, 62.5% [0.437–0.789]	0.001
Norm	22/59, 37.3% [0.250–0.509]	0.001	12/32, 37.5% [0.211–0.563]	0.001
Serum albumin				
Below norm	1/28, 3.6% [0.0009–0.183]	0.001		
Norm	22/28, 78.6% [0.590–0.917]	0.001	20/32, 65.5% [0.437–0.789]	0.001
Above norm	5/28, 17.9% [0.060–0.396]	0.019	12/32, 37.5% [0.211–0.563]	0.001

**Table 3:** Mean values of hematological parameters during the two study periods for children with CP.

Indicator	Period	N	Mean	Median	SD	*t*-Test/*p*
Hemoglobin (g/L)	1	24	126.0	129.0	8.07	t = −1.938, *p* = 0.06
	2	13	132.8	132.0	13.35	
Serum Iron (µmol/L)	1	17	10.3	10.9	4.22	t = −1.517, *p* = 0.14
	2	14	12.5	13.1	4.04	
Serum Ferritin (ng/mL)	1	23	27.3	25.1	19.56	t = 0.187, *p* = 0.853
	2	14	26.1	22.3	17.78	
Serum Albumin (g/L)	1	23	43.5	44.4	5.40	t = −2.37, *p* = 0.023
	2	15	47.1	46.5	2.71	

**Table 4:** Mean values of hematological parameters during the two study periods for children with ASD.

Indicator	Period	N	Mean	Median	SD	*t*-Test/*p*
Hemoglobin (g/L)	1	5	126.4	126.0	5.41	t = −0.607, *p* = 0.55
	2	15	128.6	130.0	7.41	
Serum Iron (µmol/L)	1	5	12.8	9.79	4.82	t = −0.261, *p* = 0.79
	2	17	13.3	13.8	3.36	
Serum Ferritin (ng/mL)	1	4	34.4	31.35	11.89	t = −0.256, *p* = 0.80
	2	17	26.1	26.2	24.16	
Serum Albumin (g/L)	1	5	46.3	45.4	2.90	t = −0.390, *p* = 0.700
	2	17	46.8	46.7	2.75	

The mean values of serum albumin in children with CP and ASD are 44.9 ± 4.82 g/L and 46.7 ± 2.72 g/L, respectively (W = 322; *p* = 0.190). Normal CRP levels were observed in all participants.

**Table 5:** Group differences in meat and fish consumption frequencies across study periods.

Consumption Type First Period	Group	N	Mean	SD	Mann–Whitney U	*p*-Value
Meat	CP	43	4.70	2.06	86.5	0.070
	ASD	7	5.86	1.86		
Fish	CP	43	1.33	0.78	88.5	0.065
	ASD	7	0.71	0.76		
Consumption Type Second Period						
Meat	CP	10	4.40	1.51	55.5	0.369
	ASD	14	3.86	1.83		
Fish	CP	11	5.00	0.00	27.5	0.002
	ASD	14	2.71	1.82		

**Table 6:** Anthropometric measurements of children with CP in the first and second periods of the study.

	Period	N	Mean	Median	SD	Independent *t*-Test/*p*
WAZ	1	33	−2.96	−3.16	1.82	t = −1.39, *df* = 45, *p* = 0.170
	2	14	−2.11	−2.63	2.08	
HAZ	1	37	−3.12	−3.33	2.07	t = −2.16, *df* = 49, *p* = 0.036
	2	14	−1.80	−1.88	1.57	
BMIAZ	1	37	−2.04	−1.72	2.25	t = −1.02, *df* = 49, *p* = 0.311
	2	14	−1.34	−1.66	1.98	

**Table 7:** Anthropometric measurements of children with ASD in the first and second periods of the study.

	Period	N	Mean	Median	SD	Independent *t*-Test/*p*
WAZ	1	16	0.385	−0.055	1.018	t = 0.919, *df* = 29, *p* = 0.366
	2	15	0.005	0.080	1.28	
HAZ	1	17	0.594	0.700	0.893	t = 1.579, *df* = 30, *p* = 0.125
	2	15	−0.051	−0.020	1.39	
BMIAZ	1	17	0.021	0.120	1.27	t = 0.152, *df* = 30, *p* = 0.881
	2	15	−0.049	0.000	1.31	

**Table 8:** Correlation analysis of anthropometric, nutritional, and biochemical markers in children with ASD and CP.

		WAZ	HAZ	BMIAZ	Serum Iron	Serum Ferritin	Hb	Serum Albumin
WAZ	Pearson’s r	—						
	*p*-value	—						
HAZ	Pearson’s r	0.852 ***	—					
	*p*-value	<0.001	—					
BMIAZ	Pearson’s r	0.766 ***	0.431 ***	—				
	*p*-value	<0.001	<0.001	—				
Serum iron	Pearson’s r	0.314 *	0.326 *	0.212	—			
	*p*-value	0.040	0.033	0.172	—			
Serum ferritin	Pearson’s r	0.127	0.168	−0.051	0.256	—		
	*p*-value	0.395	0.255	0.732	0.064	—		
Hb	Pearson’s r	0.250	0.105	0.449 **	0.316 *	0.002	—	
	*p*-value	0.094	0.483	0.002	0.025	0.991	—	
Serum albumin	Pearson’s r	0.425 **	0.374 **	0.367 **	0.338 *	0.070	0.359 **	—
	*p*-value	0.003	0.008	0.010	0.013	0.601	0.007	—

Note: * *p* < 0.05, ** *p* < 0.01, *** *p* < 0.001; “—” indicates that the correlation was not computed because the variables are identical.

## Data Availability

The data are not publicly available due to ethical restrictions and the need to protect participant confidentiality.

## Abbreviations

The following abbreviations are used in this manuscript:

ASD	Autism Spectrum Disorder
BMIAZ	Body Mass Index-for-Age Z-score
CP	Cerebral Palsy
HAZ	Height-for-Age Z-score
Hb	Hemoglobin
SF	Serum ferritin
SI	Serum iron
WAZ	Weight-for-Age Z-score
